# Discovering Microcircuit Secrets With Multi-Spot Imaging and Electrophysiological Recordings: The Example of Cerebellar Network Dynamics

**DOI:** 10.3389/fncel.2022.805670

**Published:** 2022-03-18

**Authors:** Marialuisa Tognolina, Anita Monteverdi, Egidio D’Angelo

**Affiliations:** ^1^Department of Brain and Behavioral Sciences, University of Pavia, Pavia, Italy; ^2^IRCCS Mondino Foundation, Brain Connectivity Center, Pavia, Italy

**Keywords:** multi-spot recordings, optical imaging techniques, multi-electrode arrays (MEAs), cerebellar circuit, input processing, cerebellar neurons, short-term synaptic plasticity

## Abstract

The cerebellar cortex microcircuit is characterized by a highly ordered neuronal architecture having a relatively simple and stereotyped connectivity pattern. For a long time, this structural simplicity has incorrectly led to the idea that anatomical considerations would be sufficient to understand the dynamics of the underlying circuitry. However, recent experimental evidence indicates that cerebellar operations are much more complex than solely predicted by anatomy, due to the crucial role played by neuronal and synaptic properties. To be able to explore neuronal and microcircuit dynamics, advanced imaging, electrophysiological techniques and computational models have been combined, allowing us to investigate neuronal ensembles activity and to connect microscale to mesoscale phenomena. Here, we review what is known about cerebellar network organization, neural dynamics and synaptic plasticity and point out what is still missing and would require experimental assessments. We consider the available experimental techniques that allow a comprehensive assessment of circuit dynamics, including voltage and calcium imaging and extracellular electrophysiological recordings with multi-electrode arrays (MEAs). These techniques are proving essential to investigate the spatiotemporal pattern of activity and plasticity in the cerebellar network, providing new clues on how circuit dynamics contribute to motor control and higher cognitive functions.

## Introduction

In current neuroscience research, there is growing interest to understand the functioning of neuronal ensembles, which constitute local microcircuits and are intermediate between single-cell and large-scale dynamics (Silberberg et al., 2005). Local neuronal microcircuits play a key role in brain computation by processing synaptic inputs and integrating incoming information to elaborate the neuronal discharges relayed to other brain areas.

In microcircuit functioning, individual neurons electroresponsiveness and their interactions are crucial. Thus, when investigating microcircuit activity, techniques able to acquire data from multiple single neurons simultaneously and to capture short- and long-term changes in neuronal dynamics are needed. The acquisition of this type of data is crucial to understand not only internal microcircuit dynamics but also the impact of microcircuit activity on whole brain functioning. Not surprisingly, to bridge the gap between microscale and macroscale activities, brain computational modeling is increasingly demanding information on neuronal interactions in local microcircuits. Although *in vivo* studies provide insight into biological activity underpinning behavior, *ex vivo* studies are still needed to investigate cellular mechanisms, by performing measurements that would otherwise be unfeasible in living animals or subjects.

In this review, we evaluate the recent developments in imaging and electrophysiological techniques used to perform multiple single neurons recordings on microcircuits *ex vivo*. These techniques allow now to record the activity of several cells simultaneously, monitoring their interactions and evaluating excitation-inhibition integration and synaptic plasticity. The pros and cons of multi-spot recordings are exemplified for the cerebellar cortex microcircuit. This circuit is characterized by a complex connectivity pattern (Apps et al., 2018), non-linear excitation and inhibition properties and multiple forms of synaptic plasticity that cooperate to generate the computational schemes that process incoming signals and generate the cerebellar output. The cerebellar example illustrates how the techniques reviewed here can provide powerful tools for a comprehensive assessment of the activity and connectivity in neuronal ensembles, overcoming past limitations and opening new perspectives in the modeling field (D’Angelo et al., 2016).

### What We Want: A Deeper Insight Into Microcircuit Functioning and Information Processing

**FIGURE 1:**
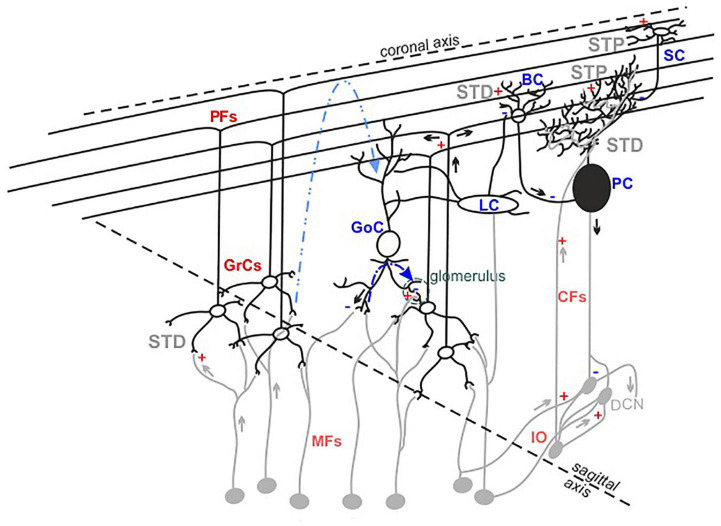
The cerebellar cortex structure and functioning. Schematic drawing of the neuronal composition of the cerebellar cortex. The colors indicate excitatory (red) and inhibitory (blue) neurons and synapses. The feedforward and feedback inhibition between GoCs-GrCs synapses are represented by the dotted lines (blue and light blue, respectively). The synapses known to express short-term potentiation (STP) or short-term depression (STD) are indicated.

It has long been assumed that the geometric and modular structure of cerebellar cytoarchitecture [schematically represented in Figure 1; Cerminara et al. (2015), D’Angelo (2018)] would be sufficient to explain microcircuit functioning. According to the Motor Learning Theory (Marr, 1969), only parallel fiber-Purkinje cell (PF-PC) and climbing fiber-PC (CF-PC) synapses would be involved in the cerebellar learning processes. However, experimental data collected over the last decades suggest that many more different cerebellar neurons are involved, indicating that the functioning of this network is more complex than anticipated (Hull, 2020).

Currently, it is recognized that the granular layer (GL) plays a central role in cerebellar processing. Mossy fibers-granule cells (MFs-GrCs) relay constitutes the cerebellar input stage, where each GrC receives four to five MFs terminals while one MF excites about 400 to 600 GrCs in a folium (Ito, 2006). MFs convey frequency-modulated bursts (Chadderton et al., 2004; Rancz et al., 2007), in different glomeruli, activating simultaneously GrCs and Golgi cells (GoCs) and triggering both the excitatory circuit and the feedforward (MFs->GoCs->GrCs) and feedback (MFs->GrCs->GoCs) inhibitory circuits. Within 5 ms after a single MFs stimulus, GrCs fire 1–2 spikes before GoCs inhibition prevails, thus limiting GrCs burst duration to a given time window (D’Angelo and De Zeeuw, 2009). The broad extent of GoCs axons exerts lateral inhibition on GrCs, determining a spatial reconfiguration of GL responses, in which excitation prevails in the center of stimulation (Solinas et al., 2010), while inhibition prevails in the surrounds (Mapelli and D’Angelo, 2007). The excitatory and inhibitory circuits also determine combinatorial operations in multiple small areas in the GL, suggesting specific local circuit topologies (Mapelli et al., 2010a). In the subsequent circuit stages, several dynamic mechanisms act in series, integrating and reconfiguring the received signals (D’Angelo, 2011; Courtemanche et al., 2013; Zhou et al., 2014) and generating the cerebellar output that is ultimately consolidated in deep cerebellar nuclei.

To further complicate the picture, several forms of synaptic plasticity occurring at multiple sites of the network contribute to shape the final output (Le Guen and De Zeeuw, 2010; De Zeeuw et al., 2011; Gao et al., 2012; D’Angelo, 2014; Hoxha et al., 2016). Long-term plasticity expressed at the MFs-GrCs synapses plays a pivotal role in the regulation of GrCs first spike delay, decreasing [long-term potentiation (LTP)] or increasing [long-term depression (LTD)] it, changing the number of spikes emitted by GrCs in the permissive time window (Nieus et al., 2006) and favoring short bursts transmission toward PCs and molecular layers (Arleo et al., 2010). Short-term plasticity, either as potentiation (STP) or depression (STD), operates a fine-tuning of incoming inputs. Repeated stimulation at the MFs-GrCs relay leads primarily to STD (Wall, 2005; DiGregorio et al., 2007; Gao et al., 2012), due to high release probability (Sola et al., 2004; Sargent et al., 2005), vesicle depletion (Saviane and Silver, 2006) and postsynaptic receptors desensitization. STP or STD can be expressed also by PFs synapses, depending on both the target neuron (PC or molecular layer interneurons) and the stimulation frequency (Carter and Regehr, 2000; Beierlein et al., 2007). PCs integrate and process stimuli supervised by the combined activation of PFs and CFs, generating the cerebellar cortex output. PFs-PCs synapses have low release probability (Isope and Barbour, 2002; Valera et al., 2012) and show prominent AMPARs-mediated facilitation (Atluri and Regehr, 1996; Häusser and Roth, 1997; Kreitzer and Regehr, 2000; Le Guen and De Zeeuw, 2010). CFs-PCs synapses mainly express STD (Dittman and Regehr, 1998) related to a high release probability and multivesicular release (Wadiche and Jahr, 2001; Foster et al., 2005). Both basket (BCs) and stellate cells (SCs) activities further modulate PCs response and are, in turn, modified by short-term dynamics. Following high frequency stimulation, PFs-BCs synapses show depression mediated by NMDARs components, while PFs-SCs synapses show facilitation (Carter and Regehr, 2000; Bao et al., 2010).

The combination of all these processes is crucial for the elaboration of incoming signals and the generation of cerebellar output (Pedroarena, 2020). Experimental investigations are ongoing and there are still open issues to explore. Single cells experiments cannot give precise indications about the role of multiple interactive elements. Conversely, multi-spot techniques prove critical, allowing a comprehensive assessment of microcircuits dynamics under physiological conditions and filling the gap between the single cell and microcircuit scale.

### What We Need: Multi-Spot Recordings

As shown above by taking the cerebellar circuit as an example, the dynamics that can be generated in a microcircuit are various and intertwined. To reconstruct these processes, beside the investigation of individual neuron properties, it is necessary to acquire data from multiple cells simultaneously to have in-depth information on their connectivity and dynamics. Recent developments in multi-spot optical imaging and new advances in electrophysiological techniques have contributed to this goal.

Optical imaging techniques use probes that bind to specific sites in neurons or to specific ions, transducing neuronal activity into changes in optical signal intensity. Over the past years, both voltage sensitive dye imaging (VSDi) and calcium imaging have been extensively developed and used in different preparations (Ebner and Chen, 1995; Baker et al., 2005; Bosman et al., 2005; Homma et al., 2009; Sepehri Rad et al., 2017), and in particular, in cerebellar slices.

In VSDi the dye binds to membrane neurons and acts as a transducer of changes in membrane potential voltage (Chemla and Chavane, 2010; Yan et al., 2012). The high temporal resolution (down to millisecond) achievable with this technique makes it an appropriate method for investigating networks activity (Hill et al., 2014; Fröhlich, 2016).

In the cerebellum, VSDi experiments were used to study the response of different circuit areas to MFs stimulation. VSDi results showed that GL performs combinatorial operations in response to MFs inputs under the control of the inhibitory circuit (Mapelli et al., 2010a). The high temporal resolution of this technique made it possible to observe the temporal evolution of the signal propagating from GL to molecular layer and the characteristics of this transmission, uncovering GL filtering and resonance properties (Mapelli et al., 2010b; Gandolfi et al., 2013; Casali et al., 2020). Furthermore, VSDi recordings revealed the impacts of long-term synaptic plasticity expression in the spatial reconfiguration of GL activity (Gandolfi et al., 2015) due to the activation of the CREB/c-Fos pathway (Gandolfi et al., 2017).

One of the major disadvantages of VSD signal is related to the non-specificity of the staining, which makes it difficult to isolate and discriminate the precise contribution of different components to the collected optical average signal (Chemla and Chavane, 2010; Chemla et al., 2017). This limitation can be partially solved by using genetically encoded voltage indicators (GEVIs) to target specific neuronal types (Bando et al., 2019; Mollinedo-Gajate et al., 2021). However, further improvements are required to increase their efficiency so that they can be used intensively (Yang and St-Pierre, 2016; Rhee et al., 2020).

Calcium imaging experiments are advantageous for identifying individual neurons and monitoring their activity, which is reflected in fluorescence changes related to variations in intracellular calcium concentration.

**FIGURE 2:**
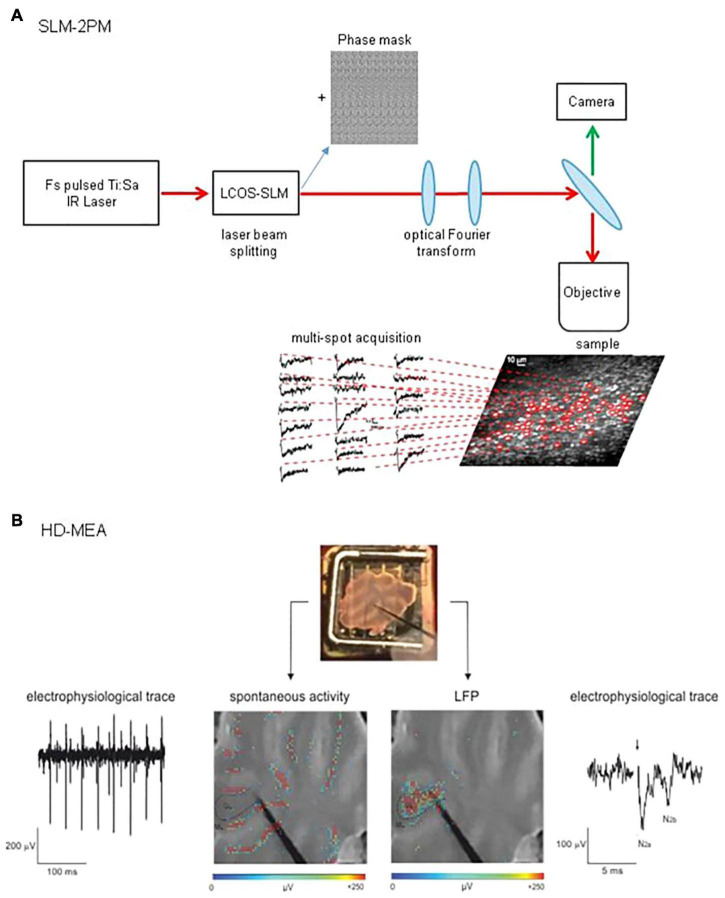
Schematic view of SLM-2PM and HD-MEA systems. **(A)** SLM-2PM. Scheme of the microscope and example of multiple stimulus-induced calcium signals acquired simultaneously from different GrCs. **(B)** HD-MEA. Top, a cerebellar slice positioned on the HD-MEA chip (stimulating electrode positioned on the MFs). Bottom left, PCs spontaneous activity (in red) can be observed selecting one of the channels in the ML. Bottom right, MFs stimulation evokes a LFP propagating through the GL; the typical N2a-N2b complex is shown in the electrophysiological trace.

To detect signals from multiple single neurons simultaneously, systems with adequate spatial resolution are needed. Two-photon microscopy fits this requirement. The integration of devices such as diffractive optical elements (DOE) (Watson et al., 2009) or spatial light modulators (SLM) (Nikolenko et al., 2008, 2013; Watson et al., 2010) into two-photon microscopes allows, by modulating the phase of a laser beam, to generate arbitrary illumination patterns and to record from different points on a sample simultaneously (Pozzi et al., 2015). In particular, two-photon SLM microscopy has been successfully implemented to study the cerebellar network. Through these experiments, it was possible to acquire calcium signals from different cell types in the network (Figure 2A). In a first study, a preliminary investigation of the circuit dynamics was conducted, which revealed sequences of neuronal activation in response to MFs stimulation (Gandolfi et al., 2014). A second work focused on the role of the inhibitory circuit in modulating GL activity before and after the expression of long-term plasticity at MFs-GrCs synapses. The data showed a combined activity of excitation and inhibition, whose cumulative activations are maintained in different spatial orientation (sagittal or coronal), and how inhibition controls the spatial expression of LTP or LTD (Casali et al., 2020).

These recent results further validate this methodology, which could be widely exploited in the future to study cerebellar processes that can only be uncovered by knowing the contribution of individual neurons in their expression. For example, it would be possible to investigate how stimuli are propagated from the input stage throughout the cortex, whether and how the expression of plasticity induced at the input stage propagates into subsequent layers, or how PCs integrate signals from PFs and molecular interneurons. The progress made in the development of VSD dyes suitable for two-photon imaging (Akemann et al., 2013; Fisher and Salzberg, 2015; Kulkarni et al., 2017; Kuhn and Roome, 2019), to be used in combination with multi-spot techniques, would allow to achieve unique spatial and temporal resolutions.

Despite the pros of the above imaging techniques, these recording modalities measure neuronal activity indirectly. The direct detection of neuronal activity is achieved using electrophysiological measurements and, in this context, extracellular recordings of signals generated by the activity of neuronal ensembles play a relevant role in the study of network dynamics. The summation of all ionic processes coming from excitable membranes into brain tissues, gives rise to an extracellular field. Currents flowing in the extracellular space around active neurons determine a voltage deflection of this electric field, which can be measured by extracellular microelectrode arrays. To retrieve information on the simultaneous activation of different portions of a network, a single electrode positioned in the extracellular space is not sufficient and a multi-electrode array (MEA) system is required. Both the relative position between the recording electrode and the cell body and their distance have a significant impact on the shape of the recorded extracellular potential. In particular, a detected spike tends to present a negative overshoot when electrodes are close to neuronal soma and a positive deflection moving down the axon (Lindén et al., 2010). Moreover, the amplitude of the detected signals decays rapidly increasing the distance between electrodes and neurons (Buzsáki et al., 2012). Besides the recording of spiking activity, electrodes can sample local field potentials (LFPs). The origin of this kind of signal is harder to discern than spikes because multiple neuronal processes concur in its generation. Synaptic activity determining the transmembrane fluxes of sodium and/or calcium ions following AMPARs and NMDARs activation is among the main contributors. In addition, cells morphology and neuronal synchrony significantly contribute to shape LFPs signals (Buzsáki et al., 2012). MEA systems allows stable recordings of extracellular signals, both as extracellular action potentials (EAPs, spikes) and LFPs from a population of neurons. For years, technical constraints have limited the size of the array and the density of electrodes, hindering the development of MEA systems able to achieve high enough spatial and temporal resolution to explore the fine-grain properties of microcircuits.

During the last decades, technological advances have led to the development of different kinds of MEAs, increasing electrode sensitivity and spatial resolution (Spira and Hai, 2013; Obien et al., 2014). The most recent improvement of MEAs technology is the high-density multi-electrode array (HD-MEA, Figure 2B), in which the increased electrodes density (e.g., 4,096 electrodes in a 2.7 mm × 2.7 mm area) enables the recording of neuronal activity at extremely high spatial and temporal resolution (Berdondini et al., 2009; Ferrea et al., 2012). This advanced technology opens new perspectives in the study of microcircuits functioning, enabling both the recording of single neurons EAPs and a comprehensive assessment of circuits dynamics in physiological conditions [e.g., acute brain slices (Emmenegger et al., 2019)].

To date, few investigations with MEAs have described cerebellar microcircuit activity. Taking advantage of the high spatio-temporal resolution of MEAs, PCs auto-rhythmic activity has been assessed simultaneously from multiple sites in the cerebellar slices (Egert et al., 2002; Frey et al., 2009), gaining detailed insights into the mechanisms underlying action potential dynamics (Bakkum et al., 2019). Cerebellar GL instead has been explored recording LFPs following MFs activation. MFs stimulation indeed determines the simultaneous firing of GrCs in a narrow time window. With extracellular electrodes, it is not possible to resolve single spikes from GrCs but it is possible to record LFPs propagating through the GL (Maffei et al., 2002; Mapelli and D’Angelo, 2007). The LFPs, characterized both pharmacologically and by means of theoretical models (Diwakar et al., 2011; Parasuram et al., 2016), show a typical N1-N2-P2 complex in which N1 corresponds to presynaptic volley activation, N2 (splitted in two components, N2a and N2b) is informative of GrCs synaptic activation and P2 represents currents returning from the molecular layer. The recording of LFPs elicited by MFs stimulation has already led to the reconstruction of excitation and inhibition controlling the GL activity (Mapelli and D’Angelo, 2007). Nevertheless, several aspects of cerebellar input processing and the role of different forms of plasticity in dynamically shaping cerebellar activity still remains unknown. Therefore, the novel high-resolution approach proposed by HD-MEAs paves the way for the reconstruction of the activity spread throughout the entire cerebellar network. Subthreshold signals would be lost together with specific information about single neuron properties, but neuronal ensembles interactions will be catched with unprecedented details compared to previous electrophysiological recordings.

## Discussion

As the knowledge of electrophysiological properties of individual neurons is refined, it becomes clear that each network element plays its own role in shaping circuit activity. Thus, only by taking into account both single and ensemble neuronal activities, it will be possible to gain a deeper insight into microcircuits functioning.

Experimental data obtained with multi-spot techniques have advanced our understanding of some functional aspects of the cerebellar cortex circuit, while other circuit dynamics deserve to be investigated. For example, a comprehensive study of cerebellar cortex responsiveness to different frequency patterns is still lacking, as the test of hypothesis about cerebellar cortex functioning (Kistler et al., 2000; D’Angelo and De Zeeuw, 2009) or the validation of model predictions of filtering properties (Casali et al., 2020; Rizza et al., 2021).

The complementarity of *ex vivo* data with those acquired *in vivo* is essential to gain a deeper understanding of microcircuit mechanisms, assessing their impact on intact brain functioning. In this perspective, technological developments in the last decade have improved the use of imaging and electrophysiological techniques in *in vivo* experiments too (Canto et al., 2016; Giovannucci et al., 2017; Yang et al., 2018; Adam et al., 2019; Barbera et al., 2019; Arlt and Häusser, 2020; Roome and Kuhn, 2020; Kim and Schnitzer, 2022). *In vivo* investigations can grasp the complexity of living organisms and are more clinically relevant than *ex vivo* ones, being closer to reality and giving the chance to explore physiological mechanisms underpinning behavior. On the other hand, *ex vivo* experiments are advantageous as they allow greater control of environmental conditions at all times, uncovering the role of different elements in the network and how their activity reverberates in the microcircuit, whose interactions are preserved following tissue slicing, although this causes the loss of some connections.

Taken together, data coming both from *ex vivo* and *in vivo* investigations of microcircuit functioning can contribute to validate the increasingly refined tools which are appearing in the modeling field to not only reproduce single cells properties but also deeply reconstruct microcircuits activations [i.e., the Brain Scaffold Builder (Casali et al., 2019; De Schepper et al., 2021)]. Overall, a combination of *ex vivo*, *in vivo* and modeling approaches could answer open questions on microcircuits secrets, getting closer to a multiscale understanding of brain functions.

## Author Contributions

MT and AM organized and wrote the manuscript, and prepared the figures. ED’A revised and contributed to the final version of the manuscript. All authors contributed to the article and approved the submitted version.

## Conflict of Interest

The authors declare that the research was conducted in the absence of any commercial or financial relationships that could be construed as a potential conflict of interest.

## Publisher’s Note

All claims expressed in this article are solely those of the authors and do not necessarily represent those of their affiliated organizations, or those of the publisher, the editors and the reviewers. Any product that may be evaluated in this article, or claim that may be made by its manufacturer, is not guaranteed or endorsed by the publisher.
